# Effectiveness of the Dual GLP-1R/GCGR Agonist Mazdutide in GSD IXc: A Case Report

**DOI:** 10.3390/jcm15208128

**Published:** 2026-10-20

**Authors:** Yingying Chen, Xueyu Zhong, Xin Guo, Yanli Xia, Cuixia Tian, Juan Zheng

**Affiliations:** 1 Department of Endocrinology, Union Hospital, Tongji Medical College, Huazhong University of Science and Technology, Wuhan 430022, China; 2 Department of Nutrition and Food Hygiene, School of Public Health, Cheeloo College of Medicine, Shandong University, Jinan 250100, China

**Keywords:** glycogen storage disease type IXc, mazdutide, GLP-1/GCGR dual agonist, hepatic fibrosis, glycemic control

## Abstract

**Background**: Glycogen storage disease type IXc (GSD IXc), caused by pathogenic PHKG2 variants leading to phosphorylase kinase deficiency, is associated with the most unfavorable prognosis among GSD IX subtypes. Patients are at markedly increased risk of progressive hepatic fibrosis and cirrhosis, for which no established disease-modifying therapies exist. Mazdutide, a dual glucagon-like peptide-1 receptor (GLP-1R) and glucagon receptor (GCGR) agonist approved for type 2 diabetes and obesity, has demonstrated beneficial effects on hepatic metabolism, steatosis, and fibrosis in preclinical studies. **Objectives**: To explore the efficacy of mazdutide for weight management, hepatic function, and glycemic control in an overweight patient with GSD IXc. **Methods**: We describe a 24-year-old woman with a 20-year history of GSD who presented with chronically elevated liver enzymes, dyslipidemia, and progressive weight gain (BMI 27.4 kg/m^2^). Genetic testing identified a homozygous PHKG2 c.765del variant, confirming the diagnosis of GSD IXc. Clinical data were collected throughout the mazdutide treatment course and follow-up, including body weight, liver stiffness, serum transaminases, lipid profiles, and glycemic parameters. Written informed consent was obtained. **Results**: After 4 months of treatment, the patient achieved a 20.6% reduction in body weight (from 85.0 to 67.5 kg). Compared with the most recent assessment performed 4 years prior, liver stiffness measurement (LSM) by transient elastography was 55% lower (29.3 to 13.1 kPa), and controlled attenuation parameter (CAP) was 18% lower (309 to 254 dB/m). Improvements in liver enzymes and partial lipid profiles were observed. Notably, the patient discontinued uncooked cornstarch (UCCS) for one month during maintenance therapy and short-term continuous glucose monitoring showed no level 2 hypoglycemia. **Conclusions**: Mazdutide may represent a promising therapeutic strategy for patients with GSD IXc accompanied by metabolic abnormalities, obesity, and hepatic fibrosis. Validation through larger cohorts and mechanistic studies is warranted.

## 1. Introduction

Glycogen storage disease type IX (GSD IX) is an inherited metabolic disorder caused by a systemic deficiency of phosphorylase kinase (PhK), a critical enzyme that regulates glycogen breakdown in multiple tissues [1]. In childhood, the clinical phenotype of GSD IX typically includes hepatomegaly, growth retardation, fasting ketosis, and hypoglycemia, all of which stem from impaired glycogenolysis and consequent disruptions in glucose homeostasis. The PhK holoenzyme is a heterotetrameric complex of α, β, γ, and δ subunits. GSD IX is classified into four primary subtypes (IXa, IXb, IXc, and IXd) according to the specific mutated subunit-encoding gene [2]. Among these, GSD IXc, caused by mutations in PHKG2 (which encodes the γ subunit), is associated with the most unfavorable prognosis. In addition to classic GSD features (fasting hypoglycemia, hepatomegaly, and growth retardation), these patients are at high risk of progressive hepatic fibrosis progressing to cirrhosis and end-stage liver disease [3]. The present patient was confirmed to have this high-risk subtype.

GSD IXc is a rare disorder, and only a limited number of adult cases have been reported. Longitudinal data on liver disease progression in adult patients remain scarce, limiting a comprehensive understanding of the natural history and long-term outcomes of this condition. Previous studies have demonstrated that this subtype can lead to severe liver injury, including cirrhosis, thereby significantly compromising long-term survival [4,5]. At present, no established therapies exist for secondary liver disease in patients with GSD IXc. Pathogenic variants in PHKG2 impair glycogenolysis, leading to pathological glycogen storage within hepatocytes. Such accumulation may trigger de novo lipogenesis via carbohydrate response element binding protein (ChREBP)-related signaling pathways. Concurrently, recurrent hypoglycemia may exacerbate peripheral lipolysis and disrupt very low-density lipoprotein (VLDL) metabolism [6,7]. Collectively, these mechanisms may contribute to hepatic steatosis, lobular inflammation, and progressive fibrosis. Notably, end-stage liver damage in GSD IXc shares pathological hallmarks with metabolic dysfunction-associated steatohepatitis (MASH).

Mazdutide is a dual agonist of the glucagon-like peptide-1 receptor (GLP-1R) and the glucagon receptor (GCGR) [8]. GCGR is expressed at relatively high levels in the liver [9]. Unlike GLP-1 receptor agonists, which primarily reduce hepatic burden indirectly through weight loss, mazdutide simultaneously activates GCGR, thereby acting directly on the liver to influence both glycogen and lipid metabolism [10]. Preclinical and clinical studies have demonstrated that mazdutide achieves substantial weight loss and potent glycemic control. Additionally, it significantly improves hepatic lipid metabolism, thereby reducing hepatic fat accumulation, inflammation, and fibrosis [11,12]. Given this dual mechanism—promoting hepatic fatty acid oxidation while ameliorating systemic metabolic disturbances—mazdutide may represent a potential pharmacological option for addressing the hepatic complications of GSD IXc, for which effective treatments are currently limited.

Given the pathophysiological rationale detailed above, the patient was commenced on mazdutide. To our knowledge, this is the first reported case of mazdutide treatment in an overweight patient with GSD IXc.

## 2. Case Presentation

Early childhood: The patient experienced recurrent episodes of hypoglycemia and hepatomegaly beginning in early childhood. At 4 years of age, clinical evaluation revealed elevated liver enzymes (ALT and AST), and a subsequent liver biopsy confirmed a diagnosis of glycogen storage disease (GSD); however, genetic subtyping was not performed at that time. Following diagnosis, the patient commenced a therapeutic regimen of uncooked cornstarch (UCCS) administered four times daily (04:30, 10:30, 16:30, and 22:30). The patient experienced myalgia during physical activity and exhibited diminished exercise tolerance, which contributed to an increased risk of weight gain. Although the frequency of hypoglycemic episodes decreased with age, occasional dizziness persisted, and routine blood glucose monitoring was not consistently performed.

Adolescence: During adolescence, the patient’s hepatomegaly gradually improved. However, chronic elevations in liver enzymes persisted, and significant dyslipidemia (elevated triglycerides and total cholesterol) developed. At 12 years of age, abdominal ultrasonography revealed fatty liver. Liver stiffness progressively increased during this period, as subsequently quantified by transient elastography.

Adulthood: At 20 years of age, transient elastography demonstrated a liver stiffness measurement (LSM) of 29.3 kPa, consistent with advanced fibrosis or cirrhosis. The patient continued prophylactic UCCS intake to maintain euglycemia, but exercise intolerance persisted, leading to progressive weight gain. Between the ages of 20 and 24 years, body weight increased from 70 kg to 85 kg, with body mass index (BMI) rising correspondingly from 22.6 to 27.4 kg/m^2^, reaching the overweight threshold. This met the clinical indications for mazdutide, defined as BMI ≥ 28 kg/m^2^, or BMI ≥ 24 kg/m^2^ in the presence of obesity-related comorbidities such as dyslipidemia. In August 2025, at 24 years of age, the patient initiated treatment with mazdutide for weight reduction, receiving an initial dose of 2 mg/week for 1 month, followed by 4 mg/week for 1 month, and then 6 mg/week for 1 month. Given the substantial weight loss and marked improvement in liver enzyme levels achieved during dose escalation, the dose was subsequently reduced to 2 mg/week for maintenance therapy to balance efficacy and tolerability and to reduce long-term treatment costs. During the first three months of mazdutide treatment, the patient used UCCS irregularly (0–2 times per day). From the fourth month onward, during one month of maintenance therapy with mazdutide at 2 mg/week, she completely discontinued UCCS.

The patient was followed in an outpatient setting. No dietary diary or validated physical activity questionnaire was available. She reported no intentional caloric restriction and no nausea, vomiting, or diarrhea. Serum albumin remained within the normal range throughout follow-up. However, normal serum albumin alone does not establish adequate nutritional status after substantial weight loss, and a comprehensive nutritional assessment was not performed.

## 3. Results

### 3.1. Genetic Testing Results and Diagnosis

**Figure 1:**
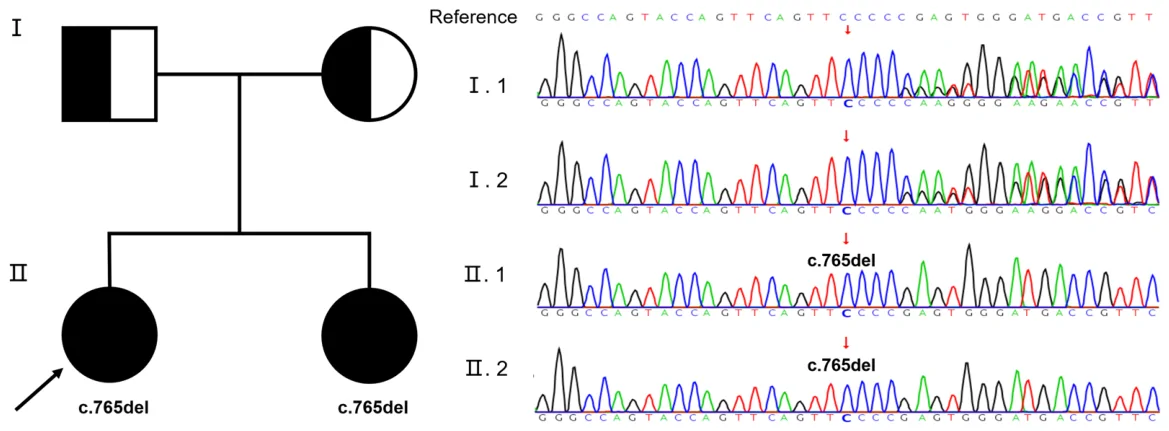
Pedigree chart of patient and sequencing results of the PHKG2 mutations. c.765del: a single-nucleotide deletion at position 765 of the PHKG2 coding sequence, resulting in a frameshift. The arrow indicates the proband.

The patient underwent genetic testing, which revealed a homozygous c.765del mutation in the PHKG2 gene. The PHKG2 c.765del variant is a frameshift mutation predicted to introduce a premature termination codon and produce a truncated, non-functional γ2 subunit. According to ACMG/AMP criteria, this variant is classified as pathogenic (PVS1 + PM2 + PP4). Family validation showed that both the patient’s father and mother were heterozygous carriers of the mutation, and the patient’s younger sister was homozygous for the c.765del mutation (Figure 1). Based on the clinical manifestations and genetic findings, the patient was ultimately diagnosed with glycogen storage disease type IXc (GSD IXc).

### 3.2. Body Weight Change

**Figure 2:**
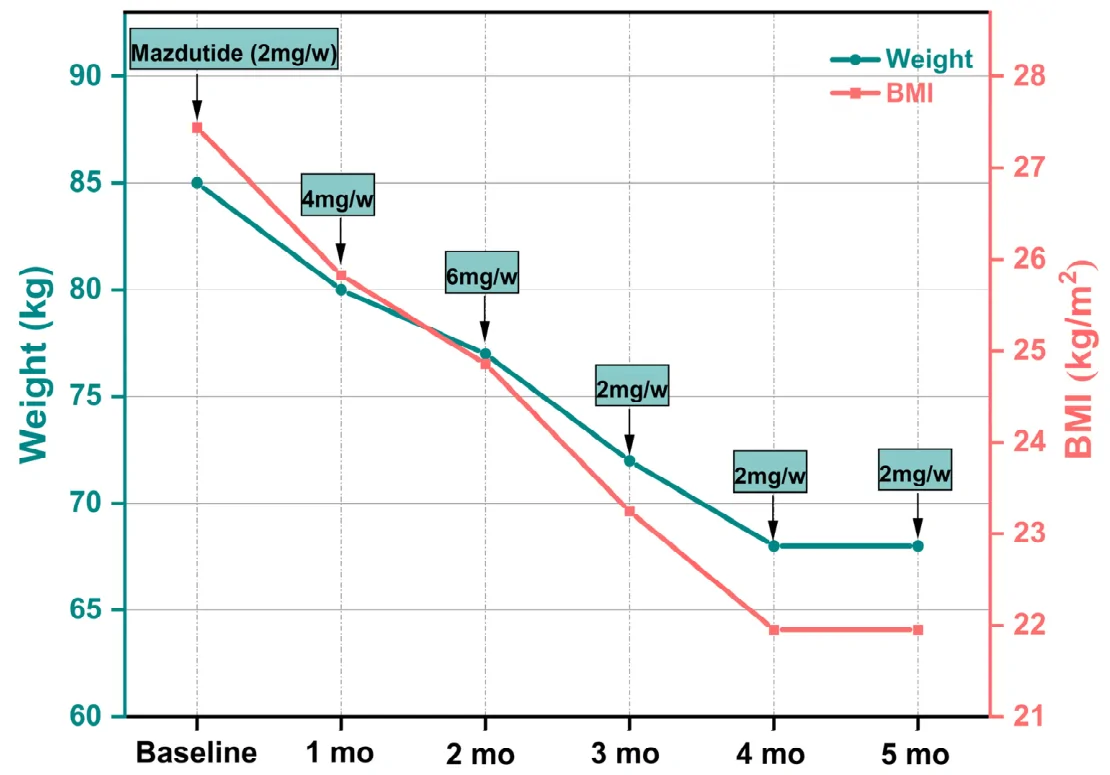
The trajectory of body weight and BMI changes in this patient during treatment. The arrows above the curve indicate the initiation time points of each dose. By the third month of mazdutide treatment, the patient’s BMI had dropped below 24 kg/m^2^, and was subsequently maintained with a 2 mg weekly dose.

The patient initiated mazdutide treatment in early August 2025 (at 24 years of age). By late November 2025 (after nearly 4 months of treatment), body weight decreased from 85 kg to 67.5 kg, a loss of 17.5 kg (a 20.6% reduction) (Figure 2).

### 3.3. Changes in Liver Status

After treatment, liver enzymes (ALT, AST) improved during follow-up and lipid changes were partial. Before treatment (at age 20), LSM was 29.3 kPa, and the Controlled Attenuation Parameter (CAP) was 309 dB/m. By November 2025 (after nearly 4 months of mazdutide treatment), LSM was 13.1 kPa, a 55% reduction., and CAP was 254 dB/m, an 18% reduction. At the 11-month follow-up, transient elastography was not performed because the patient resided in a remote area where the test was not readily accessible. Therefore, only serum-based FIB-4 was calculated (Table 1).

### 3.4. Glycemic Status

**Figure 3:**
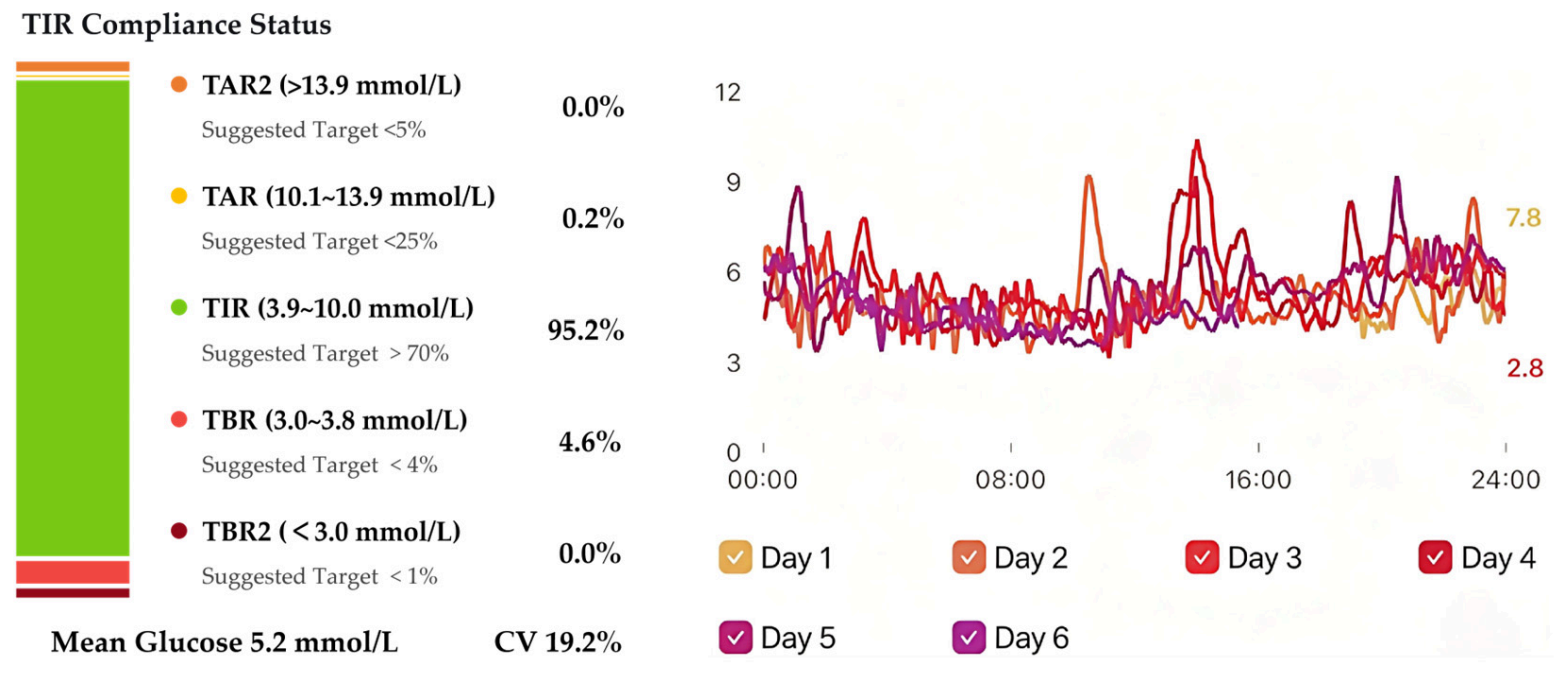
Continuous glucose monitoring data over six days during the patient’s use of mazdutide at 2 mg/week.

During the treatment period, the patient was advised to undergo continuous glucose monitoring (by Abbott) to evaluate glycemic status and the frequency of hypoglycemic episodes. During maintenance therapy with mazdutide at a dose of 2 mg/week, the patient did not take UCCS but reported no grade 2 hypoglycemia (glucose < 3.0 mmol/L) (Figure 3). The continuous glucose monitoring (CGM) parameters, including TIR, TAR, TAR2, TBR, and TBR2, mean glucose and CV are shown in Figure 3.

## 4. Discussion

Patients with GSD IXc are at a high risk of progressive liver fibrosis, which adversely impacts long-term survival. The current mainstay of treatment for this population is UCCS, which is administered primarily to maintain glycemic stability. However, effective therapeutic strategies that directly target liver fibrosis remain an unmet clinical need. Mazdutide has been approved for weight management in patients with obesity and for glycemic control in type 2 diabetes mellitus. In preclinical studies, it has been shown to effectively reduce liver fat content, improve liver inflammation, and alleviate fibrosis. Herein, we report a case of mazdutide therapy in GSD IXc, in which the patient achieved improvements in body weight, liver biochemistry, hepatic stiffness, and glycemic control.

This patient was genetically confirmed to carry a homozygous PHKG2 c.765del frameshift (truncating) variant. PHKG2 encodes the liver-specific catalytic γ2 subunit of phosphorylase kinase, and biallelic loss-of-function variants in PHKG2 cause GSD IXc. Among GSD IX subtypes, GSD IXc is associated with the least favorable prognosis and a markedly increased risk of progressive hepatic fibrosis and cirrhosis. This genotype–phenotype correlation is consistent with the patient’s clinical course, including chronically elevated liver enzymes and advanced liver stiffness by age 20. To the best of our knowledge, this represents the first reported case of mazdutide treatment in a patient with genetically confirmed GSD IXc.

The most recent liver stiffness data available prior to treatment were obtained four years earlier (at 20 years of age), when transient elastography demonstrated LSM of 29.3 kPa and CAP of 309 dB/m, indicative of advanced fibrosis. After four months of mazdutide treatment (at 24 years of age), LSM decreased to 13.1 kPa and CAP to 254 dB/m, accompanied by a 20.6% reduction in body weight (from 85 to 67.5 kg) and improvements in liver enzymes and partial lipid profiles. Additionally, the patient discontinued UCCS during maintenance therapy (2 mg/week) without grade 2 hypoglycemic episodes.

Patients with GSD IXc typically require UCCS to prevent fasting hypoglycemia due to PhK deficiency-induced impairment of glycogenolysis [13]. However, the long-term caloric burden of UCCS, combined with exercise intolerance that limits physical activity, predisposes these patients to obesity. Conventional weight loss regimens are challenging because caloric restriction can easily precipitate hypoglycemia given impaired glycogenolytic reserve, creating a therapeutic dilemma. During mazdutide maintenance therapy of 2 mg/week, UCCS was withheld for one month. During 6 days of CGM in this period, no level 2 hypoglycemia (<3.0 mmol/L) was detected. However, the time below range TBR (3.0–3.9 mmol/L) was 4.6%, indicating the occurrence of level 1 hypoglycemia. This short-term observation does not establish the safety of long-term UCCS withdrawal. Nevertheless, it represents the first reported CGM data in a patient with GSD IXc receiving a GLP-1R/GCGR dual agonist and provides a basis for future safety monitoring. Although previous studies have suggested that gluconeogenic capacity may increase with age in patients with GSD IX [1], whether mazdutide additionally promotes glycogenolysis by enhancing residual PhK activity or operates through alternative compensatory mechanisms warrants further investigation. It should be emphasized that we documented only short-term CGM data. Because the patient discontinued UCCS for only one month and CGM covered only 6 days, the glycemic safety of long-term UCCS withdrawal remains unverified. These data do not demonstrate that mazdutide permits safe withdrawal of UCCS.

The most striking observation in this case was the marked reduction in LSM. At four months after mazdutide initiation, LSM was 13.1 kPa, compared with 29.3 kPa at a historical examination performed four years earlier. However, this improvement must be interpreted with caution because no contemporaneous pre-treatment baseline LSM was available, and the four-year interval between measurements limits causal attribution. The potential influence of the natural history of GSD IXc and spontaneous fluctuation cannot be excluded. Nevertheless, given that GSD IXc typically follows a progressive course of hepatic fibrosis, and considering that the patient gained weight (BMI increased from 22.6 to 27.4 kg/m^2^), accompanied by persistently elevated liver enzymes and dyslipidemia during the four-year interval, we consider spontaneous disease remission to be unlikely. However, this possibility cannot be completely excluded in a single case.

Additionally, serial FIB-4 data provided partial longitudinal insight. FIB-4 was 1.31 approximately 14 months before mazdutide initiation, 1.91 at 2 months after initiation, and 1.06 at approximately 11 months after initiation. The increase from 1.31 to 1.91 may partly reflect progression of hepatic fibrosis before treatment, but may also be influenced by the concurrent decline in ALT during early treatment. The subsequent decrease to 1.06, into the low-risk range, was directionally consistent with the reduction in LSM. However, FIB-4 has not been validated in patients younger than 35 years and cannot replace histological assessment. Therefore, these data are presented as supportive, hypothesis-generating observations only and cannot establish a direct antifibrotic effect of mazdutide.

Notably, weight loss itself can also improve LSM, liver enzymes, and hepatic steatosis. In this patient, the substantial weight loss during mazdutide treatment means that the metabolic benefits of mazdutide observed in this case cannot be clearly separated from the effects of weight loss. We reviewed the literature and found that preclinical evidence in MASH animal models suggests that mazdutide has demonstrated superior efficacy in reducing hepatic inflammation and fibrosis [12]. Additionally, Survodutide, another GLP-1R/GCGR dual agonist, achieved significant histological improvement in a phase II trial involving patients with F2–F3 fibrotic MASH [14]. Collectively, we therefore hypothesize that GLP-1R/GCGR dual agonists may confer hepatic benefits beyond weight reduction; this hypothesis requires prospective validation with paired liver biopsy or validated fibrosis biomarkers.

Should this hypothesis be confirmed, mazdutide may represent a candidate therapeutic approach for overweight or obese patients with GSD IXc, addressing weight management, metabolic regulation, and potentially hepatic protection—an unmet need, given that these patients face a substantial risk of progressive hepatic fibrosis and current treatment remains limited to metabolic support. The favorable tolerability observed in this case is encouraging. The mechanisms underlying the observed improvements remain unclear. GCGR-mediated enhancement of hepatic fatty acid β-oxidation and suppression of de novo lipogenesis may partly contribute, but whether additional pathways—such as indirect modulation of residual PhK activity, metabolic reprogramming of hepatic stellate cells, or other unknown mechanisms—are involved requires elucidation through animal and mechanistic studies. Furthermore, the optimal dosing regimen, long-term efficacy, and potential combination strategies with uncooked cornstarch (UCCS) warrant investigation in prospective trials.

This study has several important limitations. First, immediate pre-treatment baseline LSM data were unavailable; the post-treatment improvement was compared with elastography results obtained four years earlier (at 20 years of age). The natural progression of the disease during this interval, potential effects of prior UCCS therapy, and changes in body weight may have influenced liver stiffness, precluding the establishment of a definitive causal relationship between mazdutide and LSM improvement. Second, the single-case design, absence of a control group, and relatively short follow-up period (4 months) limit the generalizability of the findings. Additionally, dietary intake and physical activity were not systematically recorded in this outpatient setting, so we cannot exclude contributions from changes in diet or activity to the observed improvements. Moreover, a comprehensive nutritional assessment after substantial weight loss was not performed, which is another important limitation. Third, although transient elastography suggested marked improvement in liver stiffness, liver biopsy (the gold standard) was not performed; therefore, this change should not be interpreted as definitive histological reversal of fibrosis. Finally, the long-term safety, optimal dosing, and sustained histological effects of mazdutide in patients with GSD IXc remain to be determined.

## 5. Conclusions

This case may provide a new therapeutic approach for the management of GSD IXc. Mazdutide may represent a novel treatment strategy for patients with GSD IXc accompanied by metabolic abnormalities, obesity, and liver fibrosis. Nevertheless, this report is only exploratory; further accumulation of clinical cases and mechanistic studies are urgently needed to validate these preliminary findings and to establish the safety and efficacy of mazdutide in this rare patient population.

## Figures and Tables

**Table 1:** Historical pre-mazdutide and follow-up laboratory and elastography data in this patient.

	Historical Pre-Mazdutide	2 m Follow-Up	4 m Follow-Up	6 m Follow-Up	11 m Follow-Up
AST, U/L	125.18 *	119.87	80	55.74	52.4
ALT, U/L	140.06 *	110.53	86	71.07	82.6
CHOL, (mmol/L)	5.88 *	6.13	5.6	6.03	-
TG, (mmol/L)	2.2 *	2.38	2.73	1.99	-
HDL-c, (mmol/L)	0.93 *	1.04	0.73	1.33	-
LDL-c, (mmol/L)	4.39 *	4.59	3.79	4.05	-
CAP, (dB/m)	309	-	254	-	-
LSM, (kPa)	29.3	-	13.1	-	-
FIB-4	1.31	1.91	-	-	1.06

* These historical pre-mazdutide data were taken as the average of the patient’s test results from the most recent four years prior to the use of mazdutide. The historical pre-mazdutide of LSM and CAP were single measurements obtained 4 years before mazdutide initiation, when the patient was 20 years old. The historical pre-mazdutide levels of FIB-4 were obtained 14 months before mazdutide initiation. Both elastography examinations were performed using FibroScan with vibration-controlled transient elastography (VCTE). The same probe type was used, and both examinations were performed in the morning after an overnight fast. In each examination, 10 valid measurements were obtained, and the IQR/median ratio was <10%. Abbreviations: AST, aspartate transaminase; ALT, alanine transaminase; CHOL, cholesterol; TG, triglyceride; HDL-c, high-density lipoprotein cholesterol; LDL-c, low-density lipoprotein cholesterol; CAP, controlled attenuation parameter; LSM, liver stiffness measurement; FIB-4, fibrosis-4 index.

## Data Availability

All authors confirm that all data supporting the findings are available from the corresponding author upon reasonable request, subject to patient confidentiality and licensing restrictions.

## Abbreviations

The following abbreviations are used in this manuscript:

ALT	alanine transaminase
AST	aspartate transaminase
CAP	controlled attenuation parameter
CGM	continuous glucose monitoring
CHOL	cholesterol
ChREBP	carbohydrate response element binding protein
FIB-4	fibrosis-4 index
GCGR	glucagon receptor
GLP-1R	glucagon-like peptide-1 receptor
GSD IXc	Glycogen storage disease type IXc
HDL-c	high-density lipoprotein cholesterol
LDL-c	low-density lipoprotein cholesterol
LSM	liver stiffness measurement
MASH	metabolic dysfunction-associated steatohepatitis
PhK	phosphorylase kinase
TG	triglyceride
UCCS	uncooked cornstarch
VLDL	low-density lipoprotein
